# *Garcinia hopii* (Clusiaceae), a new species from Bidoup Nui Ba National Park, southern Vietnam

**DOI:** 10.3897/phytokeys.77.11575

**Published:** 2017-02-28

**Authors:** Hironori Toyama, Van-Son Dang, Shuichiro Tagane, Ngoc Van Nguyen, Akiyo Naiki, Hidetoshi Nagamasu, Tetsukazu Yahara

**Affiliations:** 1 Centre for Asian Conservation Ecology, Kyushu University, 744 Motooka, Fukuoka, 819-0395, Japan; 2 National Herbarium of Vietnam, Institute of Tropical Biology, Vietnam Academy of Sciences and Technology, 85 Tran Quoc Toan Str, Dist 3, Ho Chi Minh City, Vietnam; 3 Department of Biology, Dalat University, 01 - Phu Dong Thien Vuong, Dalat, Vietnam; 4 Iriomote Station, Tropical Biosphere Research Centre, University of the Ryukyus, 870 Uehara, Taketomi-cho, Yaeyama-gun, Okinawa, 907-1541, Japan; 5 The Kyoto University Museum, Kyoto University, Yoshida Honmachi, Sakyo-ku, Kyoto, 606-8501, Japan

**Keywords:** Flora, Indochina, *matK*, *rbcL*, taxonomy

## Abstract

A new species, *Garcinia hopii* H.Toyama & V.S.Dang is described from Bidoup Nui Ba National Park, southern Vietnam. This species is similar to *Garcinia hendersoniana* Whitmore but differs from that species in having larger leaves, clustered pistillate flowers, a greater number of sterile anthers and a larger stigma of young fruits. A description, preliminary conservation assessment, illustration, photographs and DNA barcodes of the new species are provided, as well as an updated key to Garcinia sect. Hebradendron in Indochina.

## Introduction

The genus *Garcinia* L. (Clusiaceae) comprises about 260 species of usually dioecious small shrubs or trees up to 30 m tall which are common components of lowland tropical forests worldwide (Stevens 2007). The genus exhibits a remarkable diversity in floral morphology which is used for delimiting the genus and constructing its infrageneric classification (Sweeney 2008). The latest monograph, published more than a century ago by Vesque (1893), classifies 180 species into 9 sections based on floral morphology. Among the sections by Vesque (1893), Garcinia sect. Hebradendron with 19 species is distinguished from other sections by tetramerous flowers and multithecous anthers which are completely or incompletely dehiscent by a circumference slit. The most recent worldwide sectional treatment of *Garcinia* was performed by Jones (1980) in an unpublished PhD dissertation, in which she classified 31 species in Garcinia sect. Hebradendron by adding species newly described after Vesque (1893) and partially correcting the statement on anthers which are peltate with one theca dehiscing by a circumscissile slit or multithecous dehiscing by each pore. The nucleotide-based phylogenetic analysis supported the monophyly of Garcinia sect. Hebradendron (Sweeney 2008).

In Indochina, six species have been recorded in Garcinia sect. Hebradendron: *Garcinia bonii* Pit., *Garcinia elliptica* Wall. ex Wight, *Garcinia gaudichaudii* Planch. & Triana, *Garcinia hanburyi* Hook.f., *Garcinia oligantha* Merr. and *Garcinia poilanei* Gagnep. (Pierre 1880; Pitard 1910; Gagnepain 1943; Hô 1999; Dy Phon 2000; Li et al. 2007; Newman et al. 2007; Pooma and Suddee 2014). However, specimens of *Garcinia bonii* in HN (*Phuong 1535*), K (*Tsang 29824*) and P (*Butreau 39* & *Petelot 4825*) have tetramerous flowers and 4-angled stamens which are characteristic traits of Garcinia sect. Oxycarpus (Vesque 1893). Gagnepain (1943) also noted that the male flowers of *Garcinia bonii* are the same as in *Garcinia cochinchinensis* Choisy (Garcinia sect. Oxycarpus). Therefore, here we removed *Garcinia bonii* from Garcinia sect. Hebradendron.

From 2014 to 2016, botanical field surveys were carried out in Bidoup Nui Ba National Park, southern Vietnam, and a species of Garcinia sect. Hebradendron that was distinct from any of the known species was found. Here, this plant is described as a new species, *Garcinia hopii* H.Toyama & V.S.Dang, and a key for identification of all species of Garcinia sect. Hebradendron in Indochina is provided. This conclusion is based on observations of specimens in the herbaria BKF, E, HN, K, KAG, KEP, KYO, L, P, RAF, TI and VNM and specimen images on the website of JSTOR Global Plants (https://plants.jstor.org/). DNA sequences of two DNA barcode regions have also been provided; the partial genes for the large sub-unit ribulose-1,5-bisphosphate carboxylase oxygenase (*rbcL*) and maturase K (*matK*) (CBOL Plant Working Group 2009); established protocols were used to determine the sequences of these regions (Kress et al. 2009; Dunning and Savolainen 2010).

## Taxonomy

### Garcinia hopii

Taxon classificationPlantaeMalpighialesClusiaceae

H.Toyama & V.S.Dang sp. nov.

urn:lsid:ipni.org:names:

Figures 1 , 2

#### Diagnosis.

This species is similar to *Garcinia hendersoniana* Whitmore (endemic to Peninsular Malaysia) in elliptic-orbicular coriaceous leaves but differs from that species in relatively larger leaves (10‒23.5 × 6.5‒15.5 cm vs. 8–14 × 5.5–8.5 cm), clustered pistillate flowers (2–4 vs. solitary), a greater number of sterile anthers of pistillate flowers (40–64 vs. ca. 25) and a larger stigma of young fruits (4–6 mm vs. 3–4 mm in diam.).

**Figure 1.**
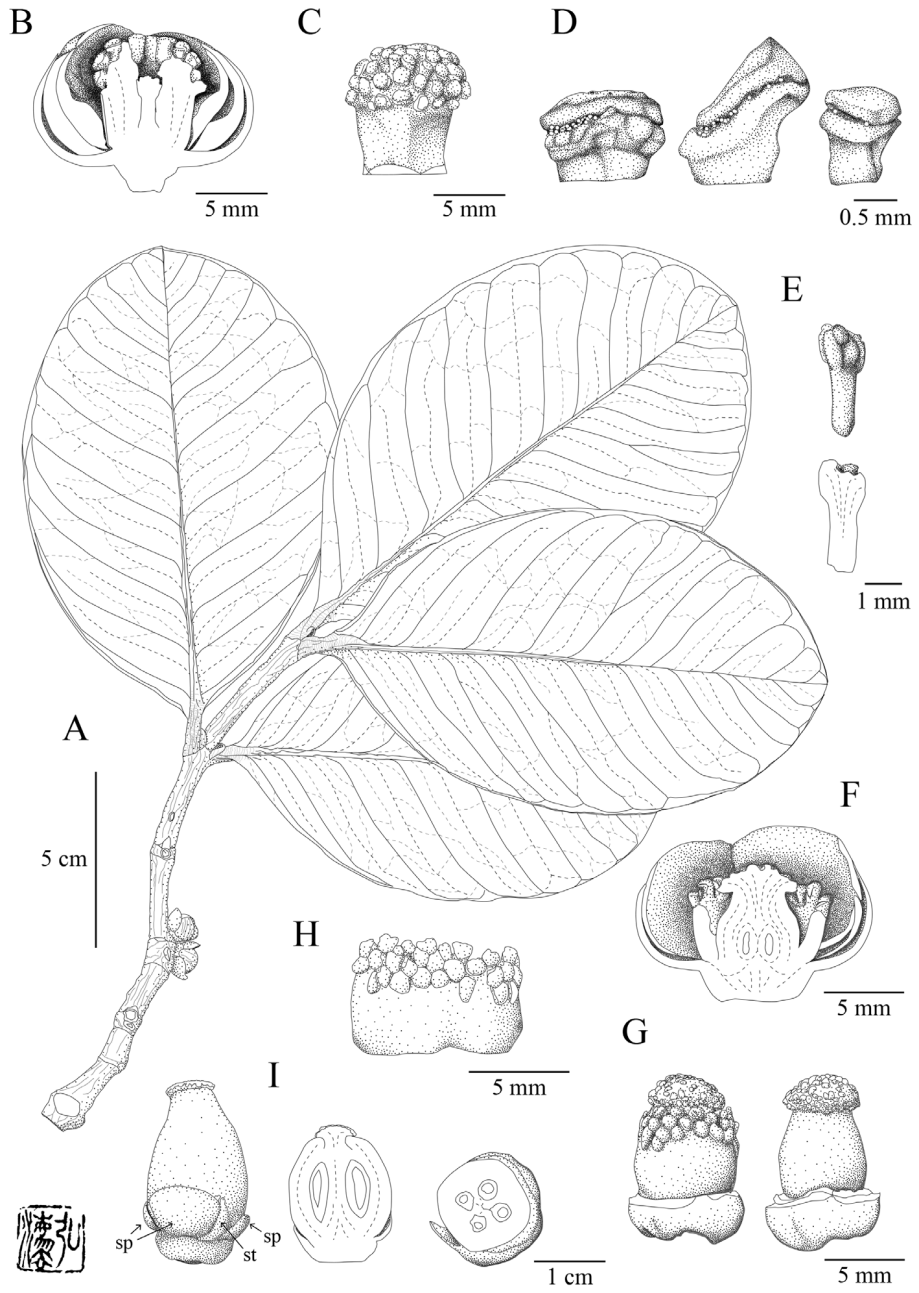
*Garcinia hopii* H.Toyama & V.S.Dang, sp. nov. **A** branch with pistillate flower **B** longitudinal section of staminate flower **C** lateral view of staminate flower, tepals removed **D** free part of stamens **E** lateral view (upper) and longitudinal section (lower) of pistillode **F** longitudinal section of pistillate flower **G** pistillate flower, tepals removed (left) and tepals and staminodes removed (right) **H** staminodes cut in half longitudinally **I** immature fruit (left) with sepals (sp) and staminodes (st) and its longitudinal (middle) and transverse (right) section. **A, F–H** from *Toyama et al. V4475* (KYO) **B–E** from *Toyama et al. V4476* (FU) **I** from *Tran & Dang dv127* (FU). Drawn by H. Toyama.

#### Type.

VIETNAM. Lam Dong Province, Bidoup Nui Ba National Park, montane evergreen forest, alt. 1781 m, 12°11.41'N, 108°42.81'E (DDM), 27 February 2016, *H. Toyama, H. Nagamasu, S. Tagane, VS. Dang, VN. Nguyen & J. Wai V4475* [female fl. & young fr.] (holotype KYO!; isotypes DLU!, FU!, NTUF!, VNM!)

**Figure 2.**
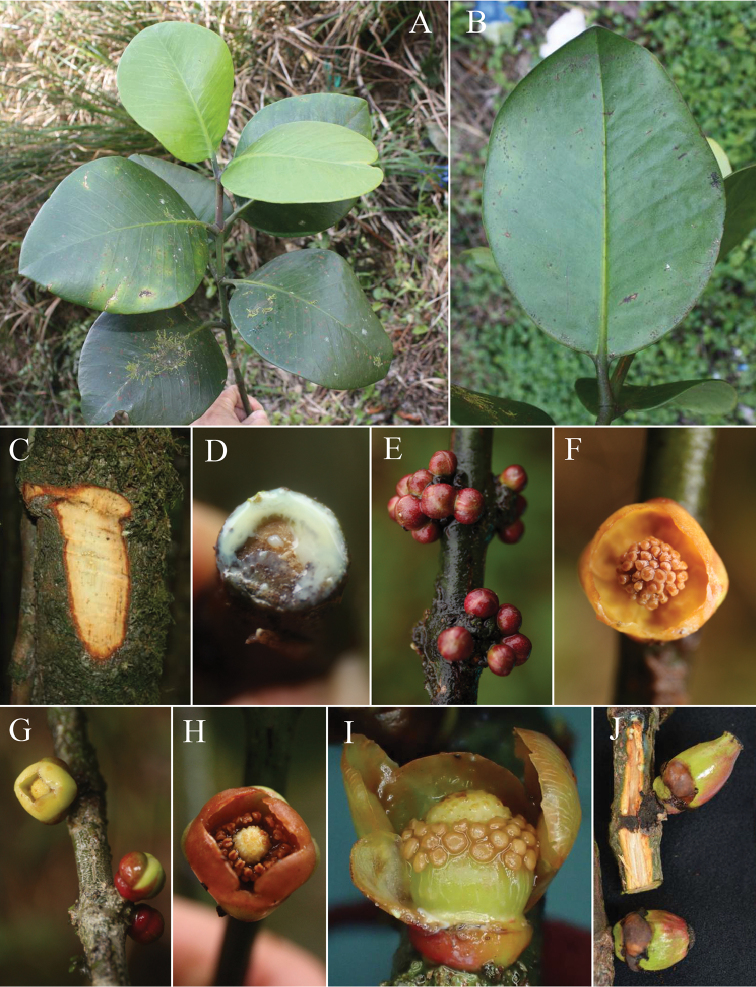
*Garcinia hopii* H.Toyama & V.S.Dang sp. nov. **A** branch with leaves **B** abaxial surface of leaf **C** trunk **D** latex **E** staminate flower buds **F** staminate flower **G** pistillate flower and buds **H** pistillate flower **I** pistillate flower, some tepals removed **J** immature fruits. **A**–**C** photographed on 22 January 2015 **E** photographed on 19 November 2014 **D, F**–**I** photographed on 27 February 2016, **J** photographed on 24 April 2015.

#### Description.

Dioecious evergreen trees up to 10 m tall, all parts glabrous; trunk pale grey-brown to brown, with pale yellow-orange latex; twigs reddish green or green and slightly tetragonous when young, turning to greenish brown or dark-brown and terete when aging, with pale yellow latex. Leaves opposite; petioles 1.0‒2.0 cm long; blade elliptic to orbicular, (4‒)10‒23.5 × (3.2‒)6.5‒15.5 cm, length/width ratio 1.2–1.9, thickly coriaceous, obtuse to rounded at base, acute to rounded at apex, margin entire, slightly recurved when dried; mid-ribs slightly prominent above and prominent below; lateral veins 10‒18 pairs, prominent and distinct on both surfaces when dried, joining into a weak intra-marginal vein that is ca. 2 mm apart from the margin; tertiary venation slightly visible on both surfaces when dried. Inflorescence of staminate flowers axillary, fascicles of (1–)2–9 flowers. Staminate flowers tetramerous; pedicels ca. 2 mm long; sepals 4, ovate-orbicular, outer sepals 6.5–9 × 7–9.5 mm, inner ones 7–9 × 8–10 mm wide, apex rounded, dark red when young, turning yellowish green when aging; petals 4, ovate-orbicular, outer petals 7–9.5 × 9–13 mm, inner ones 7–9 × 8–12 mm, thicker than sepals, apex rounded, bright yellow to yellow-orange; stamens 46–55, pharangiate, surrounding pistillode; free part of stamens 0.7–1.5 × 0.5–1 mm; free part of filaments ca. 0.5 mm long; anthers with one theca, peltate, dehiscing by a circumscissile slit; pistillode present, ca. 3.5 mm long, ca. 1 mm in diam. Inflorescence of pistillate flowers axillary, fascicles of (1–)2–4 flowers. Pistillate flowers tetramerous; pedicels ca. 2 mm long; sepals ovate-orbicular, outer sepals 6–9 × 8–10 mm, inner ones 7–8 × 9–10 mm, apex rounded, dark red when young, turning yellowish green when aging; petals ovate-orbicular, outer petals 8–9.5 × 8.5–11.5 mm, inner ones 6–8.5 × 8–10 mm, thicker than sepals, apex rounded, bright yellow or pale dark red; staminodes present, 40–64, united in a ring surrounding pistil, 5–6 × 18–22 mm when open, connate into a receptacle; free part of filaments almost sessile; pistil 5.5–10 mm long, 5–7 mm in diam.; ovary ovoid, 3–6 mm long, 4.8–7 mm in diam., 4-locular; style ca. 1–2 mm long, 3–4.5 mm in diam.; stigma convex, 2–2.5 mm long, 4–5.5 mm in diam., papillose. Young fruits (*Toyama et al. V4475*, *Tran & Dang dv127*) solitary, ellipsoid or flask-shaped, 1.1–2.0 cm long, 1.3–1.4 cm in diam., yellow green with red gradient, sepals and staminodes persistent at base, stigma persistent at apex, ca. 1 mm long, 4–6 mm in diam., slightly convex when young, turning to flat when aging; pedicels ca. 3 mm long. Mature fruits unknown. Seeds unknown.

#### Other specimen examined.

VIETNAM. Lam Dong Province, Bidoup Nui Ba National Park, 12°11'N, 108°43'E, 23 April 1997, *L. Averyanov, NQ. Binh & NT. Hiep VH4229* [female fl.] (HN!); ibid., alt. 1644 m, 12°11.21'N, 108°42.87'E (DDM), 19 November 2014, *H. Toyama, S. Tagane, VS. Dang, H. Nagamasu, A. Naiki, H. Tran, CJ. Yang, NQ. Cuong, HNP. Hieu & XN. Loi V1891* [male fl. buds] (FU!, VNM!); ibid., alt. 1644 m, 12°11.21'N, 108°42.87'E (DDM), 24 April 2015, *H. Tran & VS. Dang dv127* [male fl. & young fr.] (KYO!, VNM!); ibid., alt. 1807 m, 12°11.47'N, 108°42.78'E (DDM), 23 February 2016, *S. Tagane, H. Nagamasu, A. Naiki, VS. Dang, VN. Nguyen & J. Wai V4174* [male fl.] (DLU!, FU!, NTUF!, VNM!); ibid., alt. 1807 m, 12°11.47'N, 108°42.78'E (DDM), 27 February 2016, *H. Toyama, H. Nagamasu, S. Tagane, VS. Dang, VN. Nguyen & J. Wai V4476* [male fl.] (DLU!, FU!, NTUF!, VNM!)

#### Distribution and habitat.

*Garcinia hopii* is only known from Bidoup Nui Ba National Park, southern Vietnam. It is common in moist evergreen forests dominated by *Quercus poilanei* Hickel & A.Camus, *Neolitsea umbrosa* (Nees) Gamble, *Podocarpus neriifolius* D.Don, *Polyosma nhatrangensis* Gagnep. and *Symplocos sulcata* Kurz at alt. 1640–1810 m.

#### Phenology.

Flower buds were observed in November. Flowers were observed in February and April. Immature fruits were observed in April.

#### Etymology.

*Garcinia hopii* is named after Prof. Hop Tran, University of Science Ho Chi Minh City, who collected the flowering and fruiting specimens [*Tran & Dang dv127* (FU, VNM)].

#### Preliminary conservation status.

*Garcinia hopii* is commonly found at Hon Giao Ridge area in Bidoup Nui Ba National Park. There are many reproductive trees and the forest is well protected. Therefore, this species is assessed as Least Concern (LC) according to IUCN Red List Categories (IUCN 2012).

#### Note.

In Indochina, *Garcinia hopii* is similar to *Garcinia poilanei*, but differs from that species in having larger leaves (10‒23.5 × 6.5‒15.5 cm vs. 8–11 × 5–5.5 cm), clustered staminate flowers (2–9 vs. solitary), pistillode present (vs. absent), short pedicellate flowers (pedicels ca. 2 mm long vs. sessile) and a greater number of anthers of staminate flowers (46–55 vs. 15–18).

#### GenBank Accession No.

*Toyama et al. V1891*, LC198063 (*rbcL*), LC198064 (*matK*).

### A key to the species of Garcinia sect. Hebradendron in Indochina

**Table:** 

1	Length/width ratio of lamina > 2	**2**
–	Length/width ratio of lamina < 2	**3**
2	Lamina 5–9 × 1.5–3.5 cm; petioles 4–12 mm long; secondary veins 5–6 pairs	***Garcinia oligantha***
–	Lamina 11–14 × 3–3.5 cm; petioles 10 mm long; secondary veins 10–20 pairs	***Garcinia elliptica***
3	Pedicels 0–3 mm long in staminate flowers	**4**
–	Pedicels 10–12 mm long in staminate flowers	***Garcinia hanburyi***
4	Staminate flowers in fascicles with pedicels 2–3 mm long	**5**
–	Staminate flowers solitary, sessile; stamens 15–18; pistillode absent	***Garcinia poilanei***
5	Stamens 10–25 in staminate flowers; pistillode absent. Pistillate flowers solitary; staminodes 13–19	***Garcinia gaudichaudii***
–	Stamens 46–55 in staminate flowers; pistillode present. Pistillate flowers in fascicles; staminodes 40–64	***Garcinia hopii***

## Supplementary Material

XML Treatment for Garcinia hopii
